# Supplementary material for: Novel Driver Strength Index highlights important cancer genes in TCGA PanCanAtlas patients
Source: PeerJ. 2022 Aug 11;10:e13860. doi: 10.7717/peerj.13860 (PMC9375969; doi:10.7717/peerj.13860)
Supplement: Supplemental Information 2 [file peerj-10-13860-s002.zip › COHORTS/genes plots/detailed/2021_8_16_14_9_distribution_events_detailed_females_7.pdf]

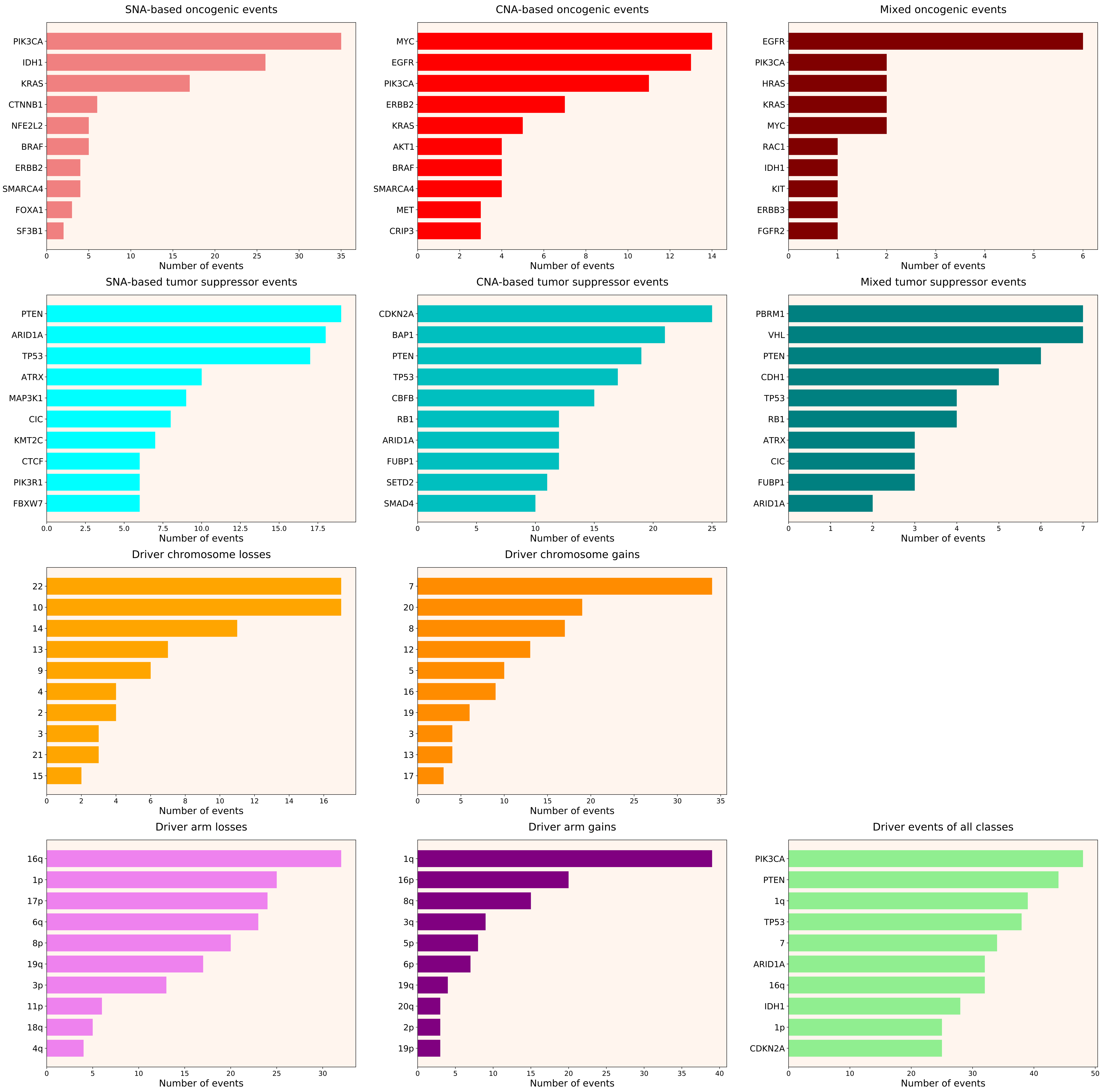
SNA-based oncogenic events
PIK3CA
IDH1
KRAS
CTNNB1
NFE2L2
BRAF
ERBB2
SMARCA4
FOXA1
SF3B1
Number of events
CNA-based oncogenic events
MYC
EGFR
PIK3CA
ERBB2
KRAS
AKT1
BRAF
SMARCA4
MET
CRIP3
Number of events
Mixed oncogenic events
EGFR
PIK3CA
HRAS
KRAS
MYC
RAC1
IDH1
KIT
ERBB3
FGFR2
Number of events
SNA-based tumor suppressor events
PTEN
ARID1A
TP53
ATRX
MAP3K1
CIC
KMT2C
CTCF
PIK3R1
FBXW7
Number of events
CNA-based tumor suppressor events
CDKN2A
BAP1
PTEN
TP53
CBFB
RB1
ARID1A
FUBP1
SETD2
SMAD4
Number of events
Mixed tumor suppressor events
PBRM1
VHL
PTEN
CDH1
TP53
RB1
ATRX
CIC
FUBP1
ARID1A
Number of events
Driver chromosome losses
22
10
14
13
9
4
2
3
21
15
Number of events
Driver chromosome gains
7
20
8
12
5
16
19
3
13
17
Number of events
Driver arm losses
16q
1p
17p
6q
8p
19q
3p
11p
18q
4q
Number of events
Driver arm gains
1q
16p
8q
3q
5p
6p
19q
20q
2p
19p
Number of events
Driver events of all classes
PIK3CA
PTEN
1q
TP53
7
ARID1A
16q
IDH1
1p
CDKN2A
Number of events
